# Molecular diversity and antimicrobial susceptibility of *Listeria monocytogenes* isolates from invasive infections in Poland (1997–2013)

**DOI:** 10.1038/s41598-018-32574-0

**Published:** 2018-09-28

**Authors:** Alicja Kuch, Anna Goc, Katarzyna Belkiewicz, Virginia Filipello, Patrycja Ronkiewicz, Agnieszka Gołębiewska, Izabela Wróbel, Marlena Kiedrowska, Izabela Waśko, Waleria Hryniewicz, Sara Lomonaco, Anna Skoczyńska

**Affiliations:** 1 National Medicines Institute, Department of Epidemiology and Clinical Microbiology, Warsaw, 00-725 Poland; 2 Nicolaus Copernicus University, Department of Genetics, Toruń, 87-100 Poland; 3 National Tuberculosis and Lung Diseases Research Institute, Department of Microbiology, Warsaw, 01-138 Poland; 4 University of Turin, Department of Veterinary Sciences, Grugliasco, 10095 Italy; 5 Istituto Zooprofilattico Sperimentale della Lombardia e dell’Emilia Romagna, Brescia, 25124 Italy; 6 US Food and Drug Administration, College Park, Maryland 20740 USA

## Abstract

The epidemiology of invasive listeriosis in humans appears to be weakly characterized in Poland, the sixth most populous member state of the European Union. We obtained antimicrobial susceptibility data, PCR-serogroups and genotypic profiles for 344 invasive isolates of *Listeria monocytogenes*, collected between 1997 and 2013 in Poland. All isolates were susceptible to the 10 tested antimicrobials, except one that was resistant to tetracycline and minocycline and harbored the *tet*(M), *tet*(A) and *tet*(C) genes. Overall, no increasing MIC values were observed during the study period. Four PCR-serogroups were observed: IVb (55.8%), IIa (34.3%), IIb (8.1%) and IIc (1.8%). We identified clonal complexes (CCs) and epidemic clones (ECs) previously involved in outbreaks worldwide, with the most prevalent CCs/ECs being: CC6/ECII (32.6%), CC1/ECI (17.2%), CC8/ECV (6.1%) and CC2/ECIV (5.5%). The present study is the first extensive analysis of Polish *L*. *monocytogenes* isolates from invasive infections.

## Introduction

*Listeria monocytogenes* is a foodborne pathogen and causative agent of infections, which occurs with different clinical presentation from self-limited gastroenteritis to severe invasive infections as sepsis or meningitis^1–4^. The groups at greatest risk of *L*. *monocytogenes* infection are pregnant women, newborns, people over 60 years of age as well as immunocompromised individuals^1,4^. Although listeriosis has low incidence (0.1–1.6 cases per 100 000 population), this disease is of great public health concern due to recurrent outbreaks and a very high fatality rate^5,6^. Clinical isolates of *L*. *monocytogenes* rarely develop antibiotic resistance^1,7,8^. However, antibiotic resistant as well as multi-resistant clinical strains of *L*. *monocytogenes* were described in 1988^1,9,10^.

*L*. *monocytogenes* is a highly heterogeneous species, which has been conventionally divided into evolutionary Lineages, serogroups, clonal complexes (CCs) and epidemic clones (ECs)^11–14^. Recently, the development of whole genome sequencing (WGS) largely improved and facilitated unambiguous identification and definition of clones and Lineages with high confidence^2^. Moreover, a recent French study on a set of 6,633 *L*. *monocytogenes* strains, suggested that this species is composed of infection-associated hyper-virulent clones (CC1, CC2, CC4 and CC6) as well as hypo-virulent food-associated clones (CC9 and CC121)^15^.

In Poland human listeriosis is an obligatory notifiable disease^16^. Cases are identified through two independent surveillance systems, a mandatory-hospital-based surveillance system run by the National Institute of Public Health-National Institute of Hygiene (NIPH-NIH) and a voluntary laboratory-based surveillance system in the National Reference Centre for Bacterial Meningitis (NRCBM). In 2008, the second system was reinforced by building a voluntary-based network (BINet) of hospital laboratories more deeply engaged in the surveillance of community-acquired invasive bacterial infections in Poland, including listeriosis. The NRCBM receives *L*. *monocytogenes* isolates, as well as data on demographic characteristics, antibiotic therapy, clinical symptoms, and the disease outcomes if already available. In the majority of cases the outcome is unknown at the time of isolate shipment. Therefore, since 2010 the NRCBM has started to actively obtain information concerning outcomes by mail or phone calls to referring microbiologists or physicians. Surveillance in Poland is highly affected by the insufficient frequency of blood culturing, which is a consequence of low adherence to the recommendations concerning blood cultures^16^.

Poland is the sixth most populous European Union (EU) country with its approximately 38.5 million of inhabitants [http://stat.gov.pl/statystyka-miedzynarodowa/porownania-miedzynarodowe]. It is one of the EU’s main suppliers of meat products and eggs, products which are also routinely exported outside of the EU. Poland is also an exporter of some processed products such slicing smoked salmon from Nordic countries [http://stat.gov.pl/obszary-tematyczne/ceny-handel/handel]. Thus, a deeper knowledge of listeriosis in Poland will help to better understand the global epidemiology of listeriosis. The aim of this study was to characterize *L*. *monocytogenes* responsible for invasive infections in Poland, by determining their antimicrobial resistance patterns, PCR-serogroups distribution and population structure. To our knowledge this is the first nation-wide analysis of Polish *L*. *monocytogenes* isolates from invasive infections.

## Results

**Figure 1:**
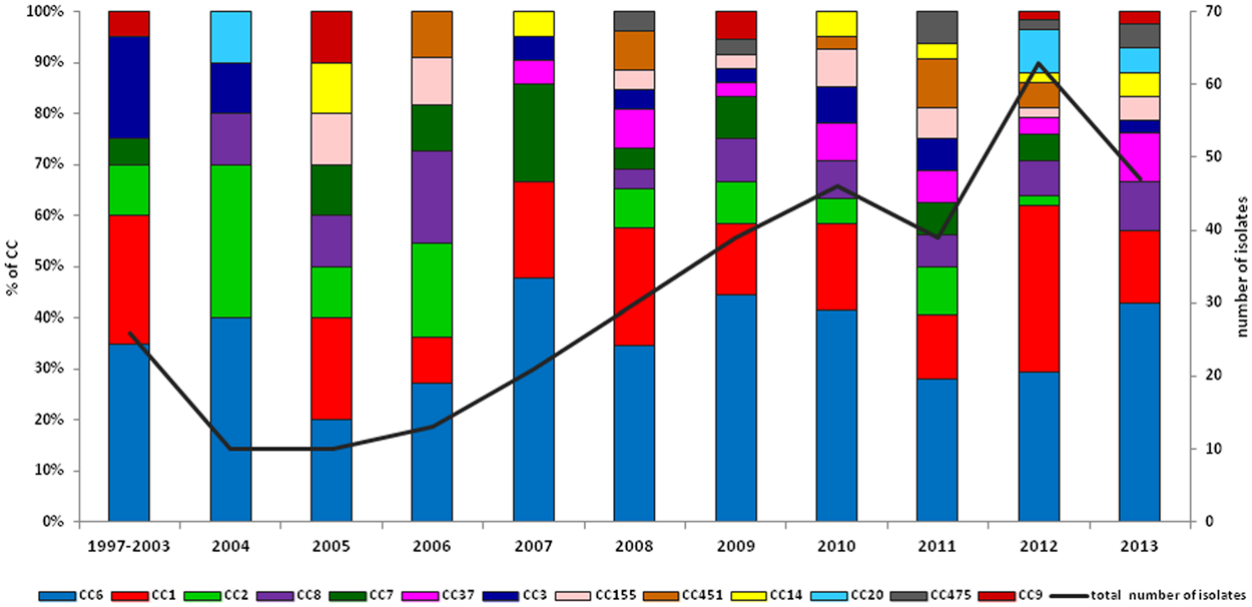
Prevalence of different clonal complexes (CCs) observed in 344 *Listeria monocytogenes* clinical isolates from Poland, 1997–2013.

**Figure 2:**
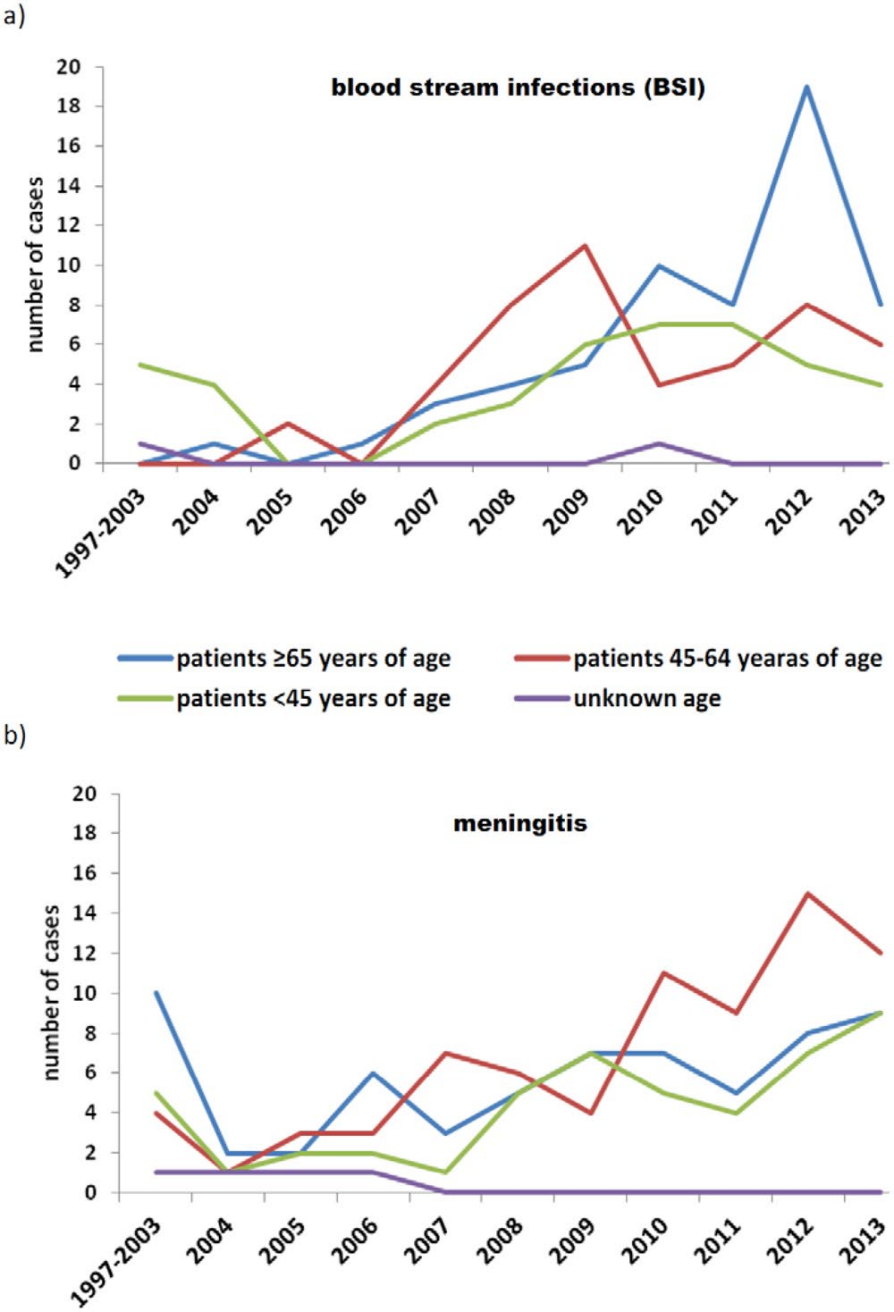
Distribution of the 344 *Listeria monocytogenes* isolates belonging to different age groups in relation to clinical presentation: blood stream infections (**a**) and meningitis (**b**); Poland, 1997–2013.

During the study period, 344 *L*. *monocytogenes* invasive isolates were collected. The isolate submission rate to the NRCBM differed throughout the years (Fig. 1). The overall incidence rate across the study period was 0.05 per 100 000 population, with the highest incidence peak in 2012 (0.17/100 000). All of patients were hospitalized (100%). Information about gender was available for 98.6% of cases, with an observed male-to-female ratio of 1.8 to 1. Age was known in 98.3% of cases, with patients ranging from 1 day to 91 years (median age 67). Specific diagnosis was available for 290 patients (84.3%). Cases with unspecific diagnosis were all categorized as bloodstream infections (BSI) since all such isolates were cultured from blood. During 2009–2013, BSIs were more common among patient ≥65 years of age in comparison to younger patients (60.8% vs. 38.9%, respectively; p = 0.005). On the other hand, meningitis was more prevalent among patients 45–64 years of age than in all other patients (60% vs. 46.9%, respectively, p = 0.04). The distribution of cases according to clinical manifestation and age is shown on Fig. 2. Among all cases there were 40 pregnancy-associated (including four from a mother-child pair). The clinical outcome was known only for 175 cases (50.9%). Overall, the case fatality rate (CFR) was 40% when only cases with known outcome were considered and 20.3% when considering all cases with unknown outcome as cured cases (n = 344) (Table 1).

**Table 1:** Characteristics of the 344 laboratory-confirmed cases of invasive listeriosis observed in Poland between 1997 and 2013.

Category		PCR-Serogroup				CFR%^d^ (n^e^)	Total (%)
		Lineage I		Lineage II			
		IVb	IIb	IIa	IIc		
Total		192	28	118	6	40 (175)	344 (100)
Gender	male	125	21	69	2	43.3 (113)	217 (63.1)
	female	65	6	47	4	34.4 (61)	122 (35.5)
	unknown	2	1	2	0	0.0 (1)	5 (1.4)
Age (years)	newborns	23	4	10	1	46.7 (15)	38 (11.0)
	1/12mo–4 y	5	1	5	0	0.0 (3)	11 (3.2)
	5–24	4	1	2	0	25.0 (4)	7 (2.0)
	25–44	17	7	10	0	25.0 (16)	34 (9.9)
	45–64	79	5	39	1	28.8 (66)	124 (36.0)
	≥65	61	10	50	3	55.7 (70)	124 (36.0)
	unknown	3	0	2	1	0.0 (1)	6 (1.9)
Clinical material	cerebrospinal fluid	106	12	51	1	26.4 (72)	170 (49.4)
	blood	83	16	64	5	48.9 (98)	168 (48.8)
	other^**a**^	3	0	3	0	60.0 (5)	6 (1.8)
Clinical presentation	meningitis	119	13	58	2	34.9 (83)	192 (55.8)
	BSI^b^	73	15	60	4	47.1 (87)	152 (44.2)
Pregnancy associated^c^		24	4	11	1	43.8 (16)	40 (100)
CFR^§^ (¶)		38.9 (95)	57.1 (7)	38.6 (70)	66.7 (3)	40 (175)	

n, Number of isolates. ^a^Rectal swab from newborn with listeriosis, sample of the retroperitoneal space and cecum tumor, pleural fluid and peritoneal fluid. ^b^BSI, blood stream infections. ^c^These cases are also counted in clinical presentation (four isolates were from mother-child pair). ^d^CFR, the overall case fatality ratio (%) was calculated taking into account only cases with known outcome. ^e^Number of cases with known outcome.

Overall, all strains were grouped into four PCR-serogroups IIa (comprising serotypes 1/2a, 3a), IIc (1/2c, 3c), IIb (1/2b, 3b), and IVb (4b, 4d, 4e). The most frequent PCR-serogroup was IVb (n = 192, 55.8%), followed by IIa (n = 118, 34.3%), IIb (28, 8.1%), and IIc (n = 6, 1.8%) (Table 1). Between 2004 and 2013 the prevalence of the different PCR-serogroups slightly fluctuated, with PCR-serogroup IVb being the dominant, except for 2011 when it was replaced by PCR-serogroup IIa. PCR-serogroup IVb was the most prevalent in meningitis (62.0% vs 38.0%, p = 0.01), in comparison to other PCR-serogroups. PCR-serogroups distribution was not related to gender or age group.

**Figure 3:**
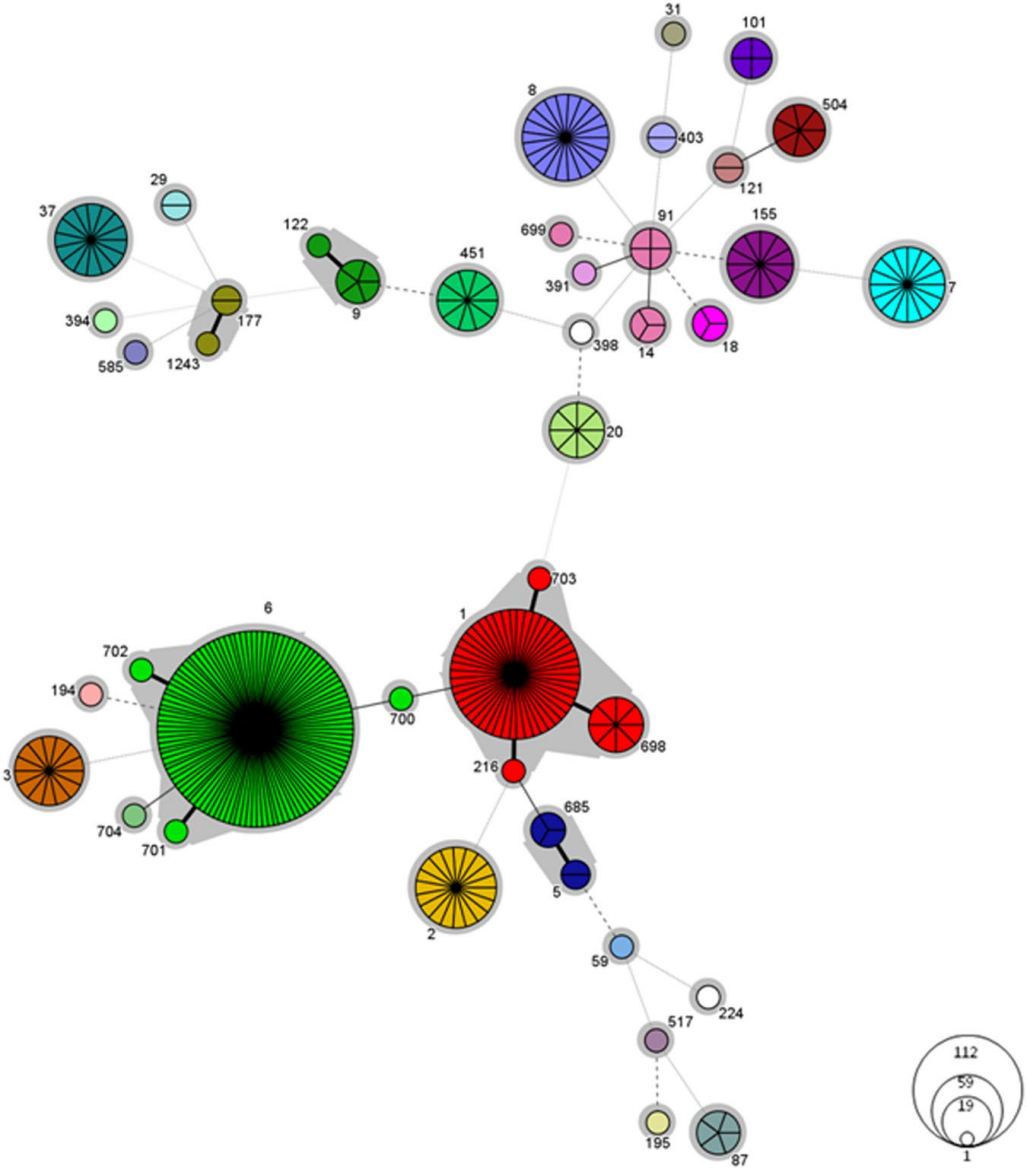
Minimum spanning tree of MLST data for 344 *L*. *monocytogenes* isolates. Each circle represents one ST and the fragment of pie chart corresponds to single isolates. The size of the circle is proportional to the number of isolates with that ST. Gray zones surrounding groups of STs represent CC. The CCs numbers are indicated by gray text for this represented by 10 and more isolates. Connecting lines infer phylogenetic relatedness in terms of number of allelic differences (thick solid, 1; thin solid, 2; dashed, 3; dotted, 4 and above).

We found 63.9% of isolates (n = 220) of Lineage I, and 36.1% (n = 124) of Lineage II (Table 2). MLST allowed the identification of 43 different STs (Simpson’s diversity index 0.984) with eight STs designated for the first time. Nineteen STs (44.2% of all STs) were represented by single isolates. Thirty CCs and two singletons (ST585 and ST704) were identified (Fig. 3). Two CCs were the most prevalent: CC6 (n = 112 isolates; 32.6%) and CC1 (n = 59; 17.2%); followed by CC8 (n = 21; 6.1%) and CC2 (n = 19; 5.5%) (Fig. 1). The remaining CCs (19%) were represented sporadically (Table 3). Hypo-virulent, food-associated clones CC9 and CC121^15^ were observed only infrequently. CFRs were calculated only for CCs represented by 10 or more isolates (Table 3). Considering only the cases with known outcome, the highest CFR was noted for CC8 (69.3%, p = 0.05) and it decreased to 42.9% if all remaining CC8 cases with unknown outcome are taken into account as cured cases.

**Table 2:** Molecular characterization of 344 *Listeria monocytogenes* clinical isolates collected from 1997 to 2013 in Poland.

Lineage n (%)	CCs (n)	CFR^a^	PCR-Serogroup (n)	EC (n)	VT (n)
I	CC6 (112)	40 (55)	IVb (112)	ECII (111)	
220 (63.9)				Non-EC (1)	**VT178** (1)
	CC1 (59)	30.3 (33)	IVb (59)	ECI (59)	
	CC2 (19)		IVb (19)	ECIV (16)	VT21 (16)
				Non-EC (3)	VT31 (2); VT**179** (1)
	CC315 (1)		IVb (1)	Non-EC (1)	VT93 (1)
	ST704 (1)		IVb (1)	ECII (1)	
	CC3 (14)		IIb (14)	ECVIII (14)	VT14 (14)
	CC5 (5)		IIb (5)	ECVI (5)	VT63 (5)
	CC87 (5)		IIb (5)	Non-EC (5)	VT8 (5)
	CC59 (1)		IIb (1)	Non-EC (1)	VT78 (1)
	CC195 (1)		IIb (1)	Non-EC (1)	**VT177**^b^ (1)
	CC224 (1)		IIb (1)	Non-EC (1)	VT124 (1)
	CC517 (1)		IIb (1)	Non-EC (1)	VT78 (1)
II					
124 (36.1)	CC8 (21)	69.3(13)	IIa (21)	ECV (21)	VT59 (21)
	CC7 (16)	20 (10)	IIa (16)	ECVII (14)	VT56 (14)
				Non-EC (2)	VT4 (1); 53 (1)
	CC37 (15)	30 (10)	IIa (15)	Non-EC (15)	VT61 (15)
	CC155 (12)		IIa (12)	Non-EC (12)	VT45 (11); 58 (1)
	CC451 (10)		IIa (10)	Non-EC (10)	VT2 (4); 140 (6)
	CC14 (8)		IIa (8)	ECIII (3)	
				Non-EC (5)	VT2 (1); VT107 (1); VT139 (3)
	CC20 (8)		IIc (8)	Non-EC (8)	VT74 (7); VT**170** (1)
	CC475 (7)		IIa (7)	Non-EC (7)	VT94 (7)
	CC101 (4)		IIa (4)	ECXI (3)	VT80 (3)
				Non-EC (1)	VT**171** (1)
	CC18 (3)		IIa (3)	Non-EC (3)	VT2 (2); VT118 (1)
	CC177 (3)		IIa (3)	Non-EC (3)	**VT169**^c^ (3)
	CC29 (2)		IIa (2)	Non-EC (2)	VT74 (2)
	CC121 (2)		IIa (2)	Non-EC (2)	VT2 (1); VT94 (1)
	CC403 (2)		IIa (2)	Non-EC (2)	VT100 (2)
	CC31 (1)		IIa (1)	Non-EC (1)	VT46 (1)
	CC89 (1)		IIa (1)	Non-EC (1)	VT4 (1)
	CC398 (1)		IIa (1)	Non-EC (1)	VT100 (1)
	CC415 (1)		IIa (1)	Non-EC (1)	VT2 (1)
	ST 585 (1)		IIa (1)	Non-EC (1)	VT141 (1)
	CC9 (6)		IIc (6)	Non-EC (6)	VT11 (6)

n, Number of isolates. CFR, the overall case fatality ratio (%) was calculated for CCs taking into account cases with known outcome represented by 10 or more isolates. ^a^In brackets number of cases with known outcome. ^b^VT177 is a SLV of VT21 (ECIV). ^c^VT169 is a single locus variant (SLV) of VT80 (ECXI). Bold type indicates new virulence types (VTs).

**Table 3:** Antimicrobials susceptibility of 344 invasive *Listeria monocytogenes* isolates collected from 1997 to 2013 in Poland.

Antimicrobials	MIC (µg/ml)									
	1997–2013 n = 344				1997–2004 n = 36	2005–2008 n = 74	2009–2010 n = 86	2011–2013 n = 148	MIC breakpoints	
	percentage of susceptible^d^	range	MIC_50_	MIC_90_	MIC_90_	MIC_90_	MIC_90_	MIC_90_	S≤	R>
Ampicillin^a^	100 (14%)	0.02–1	0.5	1	1	1	1	0.5	1	1
Penicillin^a^	100 (4.7%)	0.06–1	0.25	0.5	0.5	0.5	1	0.25	1	1
Meropenem^a^	100 (1.2%)	0.03–0.25	0.06	0.12	0.12	0.12	0.12	0.12	0.25	0.25
Erythromycin^a^	100	0.06–0.5	0.25	0.5	0.25	0.25	0.25	0.5	1	1
Co-trimoxazole^a^	100 (10%)	0.01–0.06	0.03	0.06	0.03	0.03	0.03	0.06	0.06	0.06
Levofloxacin^b^	100	0.12–1	0.5	1	0.5	0.5	0.5	1	2	2
Gentamicin^c^	100	0.03–0.5	0.06	0.25	0.06	0.06	0.06	0.5	1	1
Vancomycin^c^	100	0.25–1	0.5	0.5	0.5	0.5	0.5	0.5	2	2
Tetracycline^c^	99.5	0.12–64	0.5	1	1	1	1	0.5	1	2
Rifampicin^b^	100	0.01–0.25	0.06	0.12	0.12	0.12	0.12	0.06	0.06	0.5

n, Number of isolates. ^a^Interpretation according to the EUCAST clinical breakpoint value specific for *L*. *monocytogenes*^48^. ^b^Interpretation according to the EUCAST clinical breakpoint value for *Streptococcus pneumoniae*^48^. ^c^Interpretation according to the EUCAST clinical breakpoint value for *Staphylococcus* spp.^36,48^. ^d^In brackets % of susceptible isolates with borderline MIC breakpoint values.

PCR markers for ECs were detected in 199 isolates (57.8%): ECI (n = 59), ECII (n = 112), ECIII (n = 3) and ECV (n = 25). For the 170 isolates typed with Multi-Virulence-Locus Sequence Typing (MVLST), 33 VTs were identified, of which six were new. Among these 33 VTs, six were associated with previously described ECs (ECIV, ECV, ECVI, ECVII, ECVIII and ECXI) and represented 73 isolates (21.2%). The remaining 27 VTs, representing 97 isolates (28.2%), did not belong to any EC (Table 3). Overall, 248 (72.1%) of *L*. *monocytogenes* isolates were assigned to nine different ECs, with the most prevalent being ECII (112 isolates, 32.6%), followed by ECI (59 isolates, 17.2%). Among the 25 ECV-putative isolates, 21 were confirmed by MVSLT as VT59. The remaining 4 isolates belonged to: VT46/CC31, VT2/CC14, VT94/CC475, VT141/ST585.

**Figure 4:**
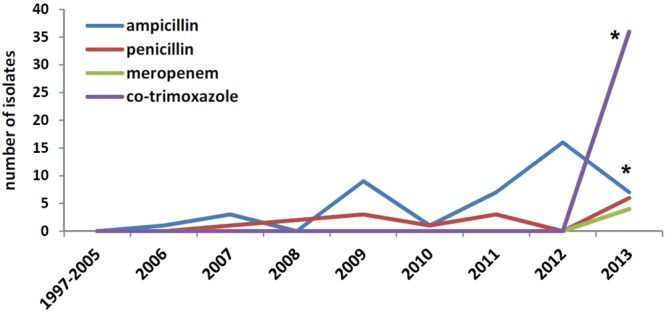
Distribution of borderline MICs (MIC at the susceptibility breakpoint) of ampicillin, penicillin, meropenem and co-trimoxazole by year, Poland 1997–2013 (* indicates p-values ≤ 0.05).

There were no differences in antimicrobial susceptibility among isolates from different periods and belonging to different PCR-serogroups. All isolates were fully susceptible to all antibiotics tested (Table 2), except one being resistant to tetracycline (MIC = 64 µg/ml) and minocycline (MIC = 8 µg/ml). Borderline MIC breakpoints were observed in *L*. *monocytogenes* strains isolated after 2006 for ampicillin (14%; p = 0.01), penicillin (4.7%; p = 0.095), meropenem (1.2%; p = 0.56) and co-trimoxazole (10%; p = 0.004) (Fig. 4). PCR screening and WGS of the tetracycline-resistant isolate identified *tet*(M) and *int* genes. WGS additionally revealed the presence of *tet*(A), *tet*(C), *sul*, *norB*, *lmo0919*, *lmo0441* and *fosX* genes. This tetracycline- and minocycline-resistant strain was isolated from CSF, belonged to PCR-serogroup IVb, Lineage I and was a member of CC2 (ST2) and ECIV (VT21).

## Discussion

The results described here, based on laboratory voluntary surveillance in Poland, revealed a remarkable increase in listeriosis incidence rates between 1997 and 2013 ranging from 0.01 in 1997 to 0.12 in 2013 per 100,000 inhabitants. The epidemiological trend observed in Poland seems to be consistent with that observed in other European countries^6,17^. However, the NRCBM received limited numbers of isolates each year before 2008 and thus the rising trend observed in Poland may be attributed only partially to an actual increase of listeriosis incidence and mostly to the enhancement of the laboratory-based surveillance system^6,16^. Interestingly, when compared with 2011 and 2013, an increase of reported listeriosis cases was observed in 2012 alongside with significant rise of CC1 contribution (data not shown). No food-borne listeriosis outbreaks were identified in Poland that year which could explain this observation^18^.

Over the last years, an increase of listeriosis cases in people 65-years-old and above has been observed in the EU and US^6,18,19^. Comparably, in our study, the median age was 67. Surprisingly, the highest number of cases was reported for patients in two age groups: i) 65-year-old and above and ii) the age group 45–64. In this respect, our findings differ from the age distribution trends observed elsewhere^3,6,18–20^. As in other studies, we noticed significantly more BSIs cases among patients 65–year-old and older compared to younger population. However, this was observed only during 2009–2013 while the Polish surveillance system was being enhanced. During the same period of observation, meningitis was significantly more common among patients 45–64 years of age, especially in 2012.

Fatality rate from invasive listeriosis is known to be high but variable, ranging from 20% to 30%, and thus the disease has been described as the most frequent cause of foodborne infection-related death in Europe^1,6,17^. In our study, the overall CFRs in cases with known outcome (40%) and in all cases (20.3%) were higher than the mean CFR in EU (15.7%)^6,17^. However, these results must be interpreted with caution due to significant differences in surveillance systems and data completeness in EU countries^17^. During the study period in Poland for example, the enhancement of a laboratory surveillance system in 2008 improved patient data completeness and available outcome data of patients increased from 31.8% to 59.8% (data not shown).

As expected, a fatal outcome was observed more frequently in patient ≥65 years of age but also in the age group 45–64. However, we also observed a high CFR (46.7%) in newborns, although outcome was known only for 38.5% cases and this was not statistically significant (p = 0.79). High fatality rates of listeriosis in newborns were also reported in Lombardy, Italy (30%), and in England (24%)^21,22^. Recently, a French study suggested that CFR might be generally higher in pregnancy-related cases than reported to date. They observed more than 80% of infected mothers experiencing major fetus or neonatal complications including death^4^. Therefore, the implementation of prevention strategies during pregnancy as well as continuous improvement of pregnant women’s awareness on listeriosis and dietary recommendations is still needed.

During the last few years, an increase in listeriosis caused by serogroup IIa was noticed in most of the European countries where this serogroup is more frequently isolated^3,15,23,24^. In our study however we did not observe an increased prevalence of PCR-serogroup IIa. The most common PCR-serogroup was IVb which accounted for more than 55% of invasive listeriosis cases. The highest frequency of PCR-serogroup IVb during the study period was fairly constant with the exception of 2011, when PCR-serogroup IIa was the most common^25^. Interestingly, most studies on non-clinical isolates from Poland (obtained from animals, meat, fresh and smoked fish) showed PCR-serogroup IIa as the most frequent, with PCR-serogroup IVb observed only sporadically^26–28^. There is only one report from Poland showing a higher percentage of PCR-serogroup IVb (31.4%) from RTE products (ready-to-eat cakes and delicatessen items, excluding meat products)^29^. It hypothesized previously that PCR-serogroup IVb may have increased virulence and may be less able to survive in foods compared to the other serogroups^6,27,30^. Our findings further confirm the strong association of PCR-serogroup IVb with invasive infections.

Most of the isolates (55.3%) analyzed in this study belonged to three of the four previously described hyper-virulent clones (i.e. CC1, CC2 and CC6)^15^. Interestingly, CC6 was the most common CC (32.6%) observed herein, in contrast to other studies worldwide reporting lower rates of this clone (approximately 3–13%)^3,12,24^. Among our CC6 isolates, 65.2% were responsible for meningitis. This is in line with a study from the Netherlands showing a significant contribution of CC6 to meningitis^31^. They observed CC6 to be associated with unfavorable outcomes in listerial meningitis but not with death^31^. Consistent with their findings, we did not observe a correlation between CC6 and meningitis fatality. Overall in our study, CC6 was the most represented clone in all types of clinical diagnosis, followed by CC1. Similarly, CC1 and CC6 were the two most common CCs related to meningitis and bacteremia in France^15^. In the same study, CC6 was also the fourth most common CC associated with pregnancy-related cases^15^.

In our study, all CC8 isolates were identified by MVLST as ECV (VT59, PCR-serogroup IIa), an EC described for the first time in the 2008 Canadian outbreak^32^. During this outbreak, CC8 was responsible for at least 57 cases with a 40% case fatality rate and continuously caused human diseases in the country between 1990s–2010s^19,32^. Even with limited data on disease outcome, our study showed that a higher fatality rate was significantly correlated with this clone (69.3%), despite all isolates apparently being sporadic and not outbreak-associated. These findings point out the need for continued enhanced surveillance of listeriosis in Poland, preferably including data on clinical outcome, as further examination will be needed to confirm our findings. For these important ECV/CC8 isolates, two genetic markers identified through comparative genomics have been proposed: chromosomal loci ML5578_229 (two primers pairs) and LM5578_1886 (one primer pair)^32^. A recent report from Italy suggested that PCR screening based on amplification of locus ML5578_229 may not be unique for ECV and therefore not sufficient for the presumptive identification of this EC^33^. In our study, PCR screening results were congruent with MVLST for all isolates of ECV only when all three above mentioned primer pairs were used. The four isolates positive for locus ML5578_229 and negative for LM5578_1886, were shown by MVLST to belong to 4 different VTs, not associated with ECV or other ECs.

In our study, CC101 was represented by four isolates of ST101. Three of them, responsible for meningitis, were typed as VT80 and belonged to ECXI. The fourth isolate (from BSI) was assigned to a new VT, namely VT171, which is a SLV (single-locus-variant) of VT80 (SNP on the 250 nucleotide of the *dal* gene fragment). The results of a multicenter study using MLST data from over 2000 *L*. *monocytogenes* isolates, identified CC101 as the only one with a clear predominance among human isolates^12^. This CC was common in the mid-1950s but it has been very rarely isolated since then^12^. This clone has recently re-emerged and it has been involved in outbreaks caused by dairy products in the US and Europe^2,14,24^. In Lombardy, Italy, CC101 was responsible for a major listeriosis outbreak linked to soft cheese in 2009–2011, and was considered as a novel EC, namely ECXI with two different MVLST profiles (VT104 corresponding to ST38, and VT80 corresponding to both ST38 and ST101)^12,14^.

Another VT, which has been recently associated with a new EC (ECVIII)^33^ is VT14, found in 14 isolates of our study (10 recovered from BSI and four from meningitis). VT14 was observed in a 1994 US multi-state outbreak due to the consumption of contaminated chocolate milk which caused at least 45 cases of febrile gastroenteritis^34^, but was also responsible for sporadic cases of listeriosis in Lombardy (2008–2013)^33^.

The correspondence in phylogenetic clustering and discriminatory power between CCs as determined by MLST and ECs as determined by MVLST has been demonstrated^11^. However, the definition of EC “*a group of isolates that are genetically related and have been implicated in temporally- and geographically-unrelated outbreaks*”^34^, assumes that *L*. *monocytogenes* strains differ in their potential to cause outbreaks. In contrast, the definition of CC is framed in terms of evolutionary biology, as “*group of isolates that descended from a common ancestor and accumulated differences mainly through mutations”*^35^, and makes no reference to the involvement in epidemics. In this respect, we believe that the characterization based on ECs may be more informative when evaluating the epidemiology of clinical cases.

Because of its severity and associated high fatality, invasive listeriosis requires an effective antimicrobial therapy, which usually involves either ampicillin or penicillin, or meropenem and co-trimoxazole^1,4^. All Polish *L*. *monocytogenes* isolates were susceptible to the antibiotics tested, with the exception of one isolate resistant to tetracycline and minocycline. Our results are comparable with observations from other countries and confirm that the frequency of acquired resistance in isolates from human listeriosis cases is low^7,8,36^. Conversely, resistance is more commonly observed in animal and food isolates^26,37,38^. As suggested by Hansen *et al*., a possible explanation as to why resistance to antibiotics is not a problem in human clinical isolates of *L*. *monocytogenes* is that resistant bacteria appear in environments where large amounts of antibiotics are used (e.g. animal farms or hospitals), and since *L*. *monocytogenes* is not a common inhabitant of patients in hospitals, it is consequently seldom exposed to antibiotics^36^. During the 17-year span of our study, MIC values for antimicrobials did not change significantly, as also seen in other countries^4,7,23,36^. However, some isolates showed borderline MIC breakpoint values, particularly for ampicillin, which is the drug of choice in the management of neuro-listeriosis^4,7^. The borderline MIC breakpoints were observed after 2006, however this observation was significant only for ampicillin and co-trimoxazole. Our finding underlines the necessity for active and continuous antimicrobial resistance surveillance for early detection of any shift in the antimicrobial susceptibility, which could limit therapeutic options of listeriosis.

As mentioned above, we found only one strain resistant to tetracycline and minocycline, suggesting ribosome protection mechanism, which was confirmed by the detection of *tet*(M) and integrase associated with the *Tn916*–*Tn1545* transposon genes. These transposons are widespread in enterococci and streptococci, and therefore their presence in *L*. *monocytogenes* is likely due to the acquisition of genetic information originating from these bacteria^9,10^. Additionally, the tetracycline-resistant strain carries *tet*(A) and *tet*(C) determinants, which are responsible for activation of efflux pumps^10^. Coexistence of these two mechanisms in the same strain could contribute to the high MIC value observed for tetracycline (64 µg/ml). *tet*(M) is the most often detected determinant among tetracycline-resistant isolates of various origins^8–10^. Conversely, *tet*(A) and *tet*(C) have been found sporadically in *L*. *monocytogenes* and mostly in isolates from animals, food and food-processing plants^10,37,38^. To the best of our knowledge, this is the first study reporting coexistence of *tet*(M), *tet*(A) and *tet*(C) genes in a human isolate from meningitis. Additionally, WGS analysis of the strain revealed the presence of *sul* (sulfonamides), *norB* (quinolone) and *fosX* (fosfomycin) resistance genes, but their presence did not correlate with phenotypic resistance (data not shown), except for fosfomycin, to which *L*. *monocytogenes* is considered to be naturally resistant^39^. However, in light of Scortti *et al*. study, *Listeria* isolates resistant to fosfomycin *in vitro*, are in fact susceptible to the antibiotic during infection (*in vitro-in vivo* paradox)^40^.

The present study is the first extensive analysis of *L*. *monocytogenes* isolates from Poland from human invasive infections using molecular techniques, including MLST and MVLST, showing predominance of PCR-serogroup IVb isolates in invasive listeriosis. We identified CCs and ECs previously involved in the outbreaks worldwide, allowing the inclusion of Polish data into the international database to track the spread of invasive strains and clones of *L*. *monocytogenes*, and contributing to the understanding of the global epidemiology of listeriosis.

## Materials and Methods

### Country background

Estimates of the national census for 31st December of every year were used as the denominator for the calculation of annual incidence rates. On average, the total Polish population was 38,276,946 during the duration of the study [Polish Central Statistical Office; http://stat.gov.pl/obszary-tematyczne/roczniki-statystyczne/]. A listeriosis case for laboratory reporting was defined as isolation of *L*. *monocytogenes* from a normally sterile body site of a patient with clinical symptoms of infection. Finally, all cases were grouped into meningitis or bloodstream infections (BSI). Listerial meningitis was defined by the isolation of *L*. *monocytogenes* from cerebrospinal-fluid or the clinical diagnosis of meningitis with *L*. *monocytogenes* isolated from blood. A pregnancy-associated listeriosis case was defined by isolation of *L*. *monocytogenes* from a pregnant woman or newborn (age <28 days) with meningitis or BSI. For all cases, only one isolate per patient was included in the study.

### Bacterial identification and PCR-serogrouping

The study encompassed *L*. *monocytogenes* isolates obtained during laboratory surveillance between January 1997 and December 2013 conducted by the NRCBM. Species identification was confirmed biochemically (API®Listeria or Vitek-2 System; BioMerieux, France) and by PCR amplification of the *hly*, *iap*, and *lmo2234* genes^41,42^. Identification of the *L*. *monocytogenes* PCR-serogroups was made by multiplex PCR^42–44^.

### Molecular characterization of isolates

The isolates were categorized into Lineages using a multiplex PCR method^45^. Sequence types (STs) were identified for all isolates either by MLST (n = 293)^46^ or were extracted from the WGS data (n = 51) obtained as a part of the European *Listeria* Typing Exercise (ELiTE WGS) organized by the European Centre for Disease prevention and Control–ECDC^47^. Raw sequence data of these 51 isolates have been deposited at the European Nucleotide Archive (Project ID PRJEB26063) (Table 4).

**Table 4:** Collection date, sequence type (ST), BioSample IDs and accession numbers for the 52 strains of *L*. *monocytogenes* analyzed with WGS. Sequences were submitted to the European Nucleotide Archive (ENA)/GenBank.

Isolate ID	Collection Date	ST	NCBI BioSample	ENA/GenBank accession #
356/04	2004	2	SAMN09692013	QPLL00000000.1
28/11	2011	2	SAMEA4587137	ERR2522387
902/11	2011	6	SAMEA4587140	ERR2522390
4492/11	2011	6	SAMEA4587144	ERR2522394
4633/11	2011	6	SAMEA4587146	ERR2522396
4853/11	2011	2	SAMEA4587147	ERR2522397
5169/11	2011	6	SAMEA4587152	ERR2522402
5170/11	2011	6	SAMEA4587153	ERR2522403
5440/11	2011	3	SAMEA4587155	ERR2522405
9259/11	2011	6	SAMEA4587161	ERR2522411
3496/12	2012	451	SAMEA4587162	ERR2522412
10203/11	2011	1	SAMEA4587164	ERR2522414
378/12	2012	6	SAMEA4587165	ERR2522415
3772/12	2012	7	SAMEA4587173	ERR2522423
3941/12	2012	1	SAMEA4587175	ERR2522425
4093/12	2012	1	SAMEA4587176	ERR2522426
4244/12	2012	698	SAMEA4587177	ERR2522427
4832/12	2012	6	SAMEA4587183	ERR2522433
4691/12	2012	6	SAMEA4587184	ERR2522434
5079/12	2012	1	SAMEA4587185	ERR2522435
5054/12	2012	6	SAMEA4587188	ERR2522438
5227/12	2012	121	SAMEA4587191	ERR2522441
5348/12	2012	6	SAMEA4587193	ERR2522443
5821/12	2012	6	SAMEA4587244	ERR2522450
5827/12	2012	6	SAMEA4587245	ERR2522451
6087/12	2012	6	SAMEA4587246	ERR2522452
5981/12	2012	8	SAMEA4587248	ERR2522454
5921/12	2012	1	SAMEA4587249	ERR2522455
6301/12	2012	7	SAMEA4587252	ERR2522458
6334/12	2012	1	SAMEA4587253	ERR2522459
6547/12	2012	6	SAMEA4587254	ERR2522460
6836/12	2012	1	SAMEA4587257	ERR2522463
6906/12	2012	6	SAMEA4587259	ERR2522465
7813/12	2012	6	SAMEA4587263	ERR2522469
78/13	2013	1	SAMEA4587264	ERR2522470
322/13	2013	698	SAMEA4587265	ERR2522471
618/13	2013	1	SAMEA4587267	ERR2522473
941/13	2013	6	SAMEA4587268	ERR2522474
1767/13	2013	6	SAMEA4587274	ERR2522480
3015/13	2013	6	SAMEA4587280	ERR2522486
3011/13	2013	1	SAMEA4587281	ERR2522487
3222/13	2013	6	SAMEA4587283	ERR2522489
3778/13	2013	6	SAMEA4587285	ERR2522491
4043/13	2013	1	SAMEA4587287	ERR2522493
243/14	2013	20	SAMEA4587289	ERR2522495
4166/13	2013	6	SAMEA4587291	ERR2522497
4379/13	2013	6	SAMEA4587292	ERR2522498
4199/13	2013	37	SAMEA4587293	ERR2522499
4820/13	2013	6	SAMEA4587301	ERR2522507
5014/13	2013	8	SAMEA4587302	ERR2522508
5044/13	2013	6	SAMEA4587303	ERR2522509
5133/13	2013	8	SAMEA4587304	ERR2522510

Allele numbers, STs, CCs and antibiotic resistance genes were determined according to the *Listeria* sequence typing database [http://bigsdb.pasteur.fr/listeria/listeria.htm; last accessed 12 December 2017]. Phylogenetic analysis was carried out using BioNumerics (v7.6; Applied Maths, Sint-Martens-Latem, Belgium).

The tetracycline resistant strain was not the part of ELiTE WGS, therefore the WGS was performed with Ion Torrent PGM (Thermo Fisher Scientific, Waltham, MA, USA) and obtained reads were assembled using CLC Genomic Workbench v.8 (CLC bio, Aarhus, Denmark). This Whole Genome Shotgun project has been deposited at DDBJ/ENA/GenBank under the accession QPLL00000000. The version described in this paper is version QPLL01000000 (Table 4).

Markers for four ECs (ECI-ECIII and ECV) were screened by PCR^32,42^. Subsequently, MVLST was performed on all isolates negative for those four EC markers. Additionally, all ECV-putative isolates identified by PCR were confirmed by MVLST^42^. Overall, MVLST was carried out on 170 isolates. Allele numbers and VTs were determined according to the MVLST database [https://sites.google.com/site/MVLSTdatabase/home, last accessed 23 July 2018].

### Bacterial susceptibility testing

Antimicrobial susceptibility to the following 10 antibiotics was tested for all isolates: ampicillin, penicillin, meropenem, erythromycin, trimethoprim-sulfamethoxazole (co-trimoxazole), levofloxacin, gentamicin, vancomycin, tetracycline, and rifampicin, using the broth microdilution method as recommended by the European Committee on Antimicrobial Susceptibility Testing (EUCAST)^48^. Different clinical breakpoints were used for interpretations according to the EUCAST: those specific for *L*. *monocytogenes* were used for ampicillin, penicillin, meropenem, erythromycin, and co-trimoxazole^49^; those specific for *Streptococcus pneumoniae* were used for levofloxacin, vancomycin, tetracycline and rifampicin^36,49^. For gentamicin, interpretation was carried out according to the clinical breakpoint value for *Staphylococcus* spp.^36,49^. Finally, minocycline was tested only for the tetracycline-resistant strain, which was additionally screened by PCR for the presence of resistance and integrase genes^50,51^.

### Statistical methods

The diversity index (DI) was calculated as described by Grundmann *et al*.^52^. Chi square test or Fisher’s exact test was used to analyze the differences in frequencies; p-values ≤ 0.05 were considered significant.
